# Physiologic effects and regional ventilation of high-frequency positive-pressure ventilation using a conventional ventilator in a severe ARDS animal model associated with an inspiratory pause or recruitment maneuvers

**DOI:** 10.1186/cc12060

**Published:** 2013-03-19

**Authors:** RL Cordioli, M Park, M Amato, S Gomes, E Leite, L Azevedo

**Affiliations:** 1 Hopitaux Universitaire de Geneve, Geneva, Switzerland; 2 Hospital Sirio Libanes IEP, São Paulo, Brazil; 3 Hospital das Clinicas LIM 09, São Paulo, Brazil

## Introduction

Protective mechanical ventilation (MV) in ARDS is based on reduced stretch of pulmonary tissue, sometimes resulting in severe hypoventilation that can be avoided when using high respiratory rate. High-frequency positive-pressure ventilation (HFPPV) has not been fully explored, especially when associated with other strategies aiming to avoid hypercapnia.

## Methods

We induced ARDS in eight pigs by lung lavage with saline plus 3 hours of injurious MV with low PEEP and high driving pressure (DP). We then performed a recruitment maneuver (RM) followed by PEEP titration using the amount of alveolar collapse in electrical impedance tomography (EIT). Then stabilization during 1 hours with tidal volume (VT) at 6 ml/kg, respiratory rate (RR) 35 breaths/minute and PEEP selected with the PEEP-FiO_2 _table (ARMA study), which was kept constant during two steps of HFPPV with a RR 60: one without an inspiratory pause (HFPPV-60), and one with a pause of 30% of inspiratory time (HFPPV-60 w/P30%). In another HFPPV step, we used PEEP titrated with EIT after RM (HFPPV-60 w/RM). During each HFPPV step, VT was set to reach a PaCO_2 _of 60 ± 3 mmHg. Distribution of regional ventilation was analyzed using EIT. Equilibrium was considered if PaCO_2 _was stable (<5% of variation) for >30 minutes.

## Results

HFPPV allowed reduction in PaCO_2 _levels: 81 (77, 94) versus 60 (58, 61), 59 (58, 60), 60 (58, 61) mmHg, besides using lower VT: 5.2 (5.0, 5.9), 5.1 (4.5, 6.0), 4.7 (4.2, 5.7) and 4.8 (4.5, 5.6) ml/kg during stabilization, HFPPV-60, HFPPV-60 w/P30% and HFPPV-60 w/RM, respectively. It had no significant different results comparing HFPPV-60 with and without an inspiratory pause. HFPPV-60 w/RM allowed a better alveolar homogenization and improvement in oxygenation, shunt, dead space and DP compared with the other steps. See Table 1.

**Table 1:** Physiological variables

Variable	VT = 6, stabilization	HFPPV-60	HFPPV-60 w/RM
DP (cmH_2_O)	16 ± 2	16 ± 3	12 ± 2*
P/F ratio	95 ± 13	149 ± 60	246 ± 99*

*Tukey's *post-hoc *analysis, *P <*0.05 versus others.

## Conclusion

HFPPV with a conventional mechanical ventilator is able to maintain stable PaCO_2 _in clinically acceptable values, allowing reductions in VT. HFPPV-60 w/RM and PEEP titration using EIT allowed further physiologic benefits in a severe ARDS model.

